# Update on Salvage Options in Relapsed/Refractory Hodgkin Lymphoma after Autotransplant

**DOI:** 10.1155/2014/605691

**Published:** 2014-03-30

**Authors:** Nida Iqbal, Lalit Kumar, Naveed Iqbal

**Affiliations:** ^1^Department of Medical Oncology, Dr. B. R. A. Institute Rotary Cancer Hospital, All India Institute of Medical Sciences, New Delhi 110029, India; ^2^Department of Anaesthesia and Intensive Care Unit, Indraprastha Apollo Hospital, New Delhi 110076, India

## Abstract

Despite a high clinical success, relapse in Hodgkin lymphoma occurs in 10–30% of cases and 5–10% patients are nonresponsive to initial chemotherapy. The standard management of these patients includes high-dose chemotherapy followed by autologous stem cell transplant. However, 50% of patients ultimately relapse after autotransplant which poses a big challenge. Allogeneic stem cell transplantation offers the only chance of cure in these patients. For patients who are not candidates for allogeneic stem cell transplantation, achieving cure with other possible options is highly unlikely, and thus the treatment plan becomes noncurative. Various novel agents have shown promising results but the duration of response is short lived. A standard approach to deliver the most effective treatment for these patients is still lacking. This review focuses on the treatment options currently available for relapsed and refractory disease after autotransplant.

## 1. **Introduction**

Hodgkin lymphoma (HL) is a potentially curable lymphoma with distinct histology, biologic behaviour, and clinical characteristics. The reported five-year event-free survival ranges between 80 and 90% with combined modality chemotherapy and radiotherapy [1]. Despite the high cure rate with initial therapy, approximately 5% to 10% of patients have refractory disease, and 10% to 30% patients relapse after an initial complete response [2]. Autologous stem cell transplantation (ASCT) is the standard of care for patients with relapsed HL [3]. About half of all patients undergoing ASCT are rescued and definitely cured by such an approach, but the outcome of patients relapsing or refractory to second-line chemotherapy and ASCT is dismal, with a median survival of less than three years [4]. One of the most important and widely accepted prognostic factors for patients undergoing ASCT appears to be chemosensitivity at relapse, with patients responding to second-line chemotherapy and having a much better outcome than patients with refractory disease, whose relapse rate approaches 80% in some published series [5, 6]. In the functional imaging with positron emission tomography (PET) era, PET positive response at the end of induction therapy has been found to be the worst predictor of outcome [7, 8].

There are a few published literatures on the treatment options of patients with RR-HL after autotransplant. This paper summarizes the current available treatment modalities in these patients with emphasis on novel drugs.

## 2. **Diagnosis of RR-HL**

A diagnostic rebiopsy should be considered to confirm relapse or progressive disease if the primary diagnosis was not clear and if the relapse is late (beyond 3–5 years of therapy) or unusual in pattern and in PET positive lesions whenever feasible.

## 3. **Salvage Options after ASCT**

These include radiotherapy, second ASCT, allogeneic stem cell transplant (Allo-SCT), monoclonal antibodies, chemotherapeutic drugs, and novel agents [9, 10].

## 4. **Radiotherapy**

A significant number of patients who relapse after stem cell transplant do so in previously involved sites and may present with disease that could be encompassed in a radiation field. This strategy appears most beneficial in those who present with Ann Arbor stage I or II disease at relapse, without B symptoms, and no extranodal disease. Josting et al. reported 5-year freedom-from-treatment failure (FFTF) of 28% in patients receiving either extended-field or involved-field radiotherapy [11]. Involved-field radiation is an important option when recurrent disease extends beyond previously unirradiated lymph nodes. Radiation in a prior radiation field should be considered if tissue tolerance allows; however there is little information to support this.

## 5. **Second Autologous Transplant**

This option seems to be feasible for patients who relapse >1 year after the initial transplant.

A recent report from the Center for International Blood and Marrow Transplant Research (CIBMTR) on 40 patients undergoing second transplants included 21 patients with HL: outcomes for patients relapsing within 12 months of the first transplant were very poor, but for those with relapse >3 years, progression-free survival (PFS) and overall survival (OS) were 25% and 38%, respectively [12].

## 6. **Allogeneic Stem Cell Transplantation**

Allo-SCT offers the only chance of cure for suitable patients after failed ASCT; however selecting the best conditioning regimen is still controversial. Myeloablative strategies achieve cure in some patients, but at the cost of high transplant-related mortality (TRM), whereas reduced-intensity conditioning (RIC) regimens are associated with high posttransplant relapse rates.

A report from the International Bone Marrow Transplant Registry of 114 patients with lymphoma undergoing myeloablative allogeneic transplants reported a rate of disease progression at 3 years of 52% and TRM of 22%. This translated to a relatively disappointing 3-year PFS of 25% and OS of 33%. With further followup it was found that 5-year disease-free survival (DFS) and OS were 5% and 24%, respectively [13]. Based on the assumption of allogeneic graft versus lymphoma (GvL) effect, reduced-intensity conditioning was introduced which resulted in a decreased cumulative incidence of nonrelapse mortality (NRM) ranging from 11% to 13%. Nevertheless, survival outcomes were relatively unchanged, as approximately 50% of all patients undergoing allogeneic SCT after RIC relapsed [14, 15].

Sarina et al. published the results of a retrospective multicenter study on 185 relapsed/refractory HL patients. In this study, outcomes were correlated with donor availability. A total of 122 of patients (66%) had a suitable donor. The patients from the “donor group” experienced improved 2-year OS and PFS as compared with those from the “no donor group” (OS: 66% versus 42%, PFS: 39% versus 14%, *P* < .001). The 2-year NRM rate for the transplanted patients was 13% [16].

Peggs et al. investigated the role of* in vivo* T depletion in 67 relapsed/refractory HL patients, mostof whom had previously undergone ASCT. They used afludarabine/melphalan (FluMel) regimen either with or without alemtuzumab. The cumulative TRM rate at 2 years was 7% in the alemtuzumab cohort and 29% in the “FluMel only” group. Nevertheless, both the 3-year cumulative incidence of relapse (54% versus 44%) and 3-year PFS (43% versus 25%) were higher in patients with the FluMel/alemtuzumab regimen. In addition, there was a trend to longer duration of responses in the alemtuzumab cohort (median, 33 months versus <12 months).* In vivo *T-cell depletion might contribute to better survival outcomes [17].

Although the feasibility of RIC Allo-SCT has improved, there is still a lack of durable response and the high relapse or progression rates remain unsolved problems. The need of time is to standardize the indication and the time point of allogeneic SCT in therapeutic algorithms of these patients.

## 7. **Alternative Strategies**

For patients relapsing after allogeneic SCT, or those not suitable for allogeneic SCT, alternative therapeutic approaches include the use of monoclonal antibodies, conventional chemotherapeutic drugs, or novel agents (Table 1).

### 7.1. Monoclonal Antibodies

The monoclonal antibodies that have been studied in relapsed/refractory HL target CD30, CD20, and CD25.

### 7.2. Targeting CD30: Brentuximab Vedotin

Brentuximab vedotin is an antibody-drug conjugate consisting of a chimeric anti-CD30 monoclonal antibody. In a large phase II single arm multicenter study, 102 heavily pretreated patients who relapsed after ASCT were treated using the MTD dose of 1.8 mg/kg administered every 3 weeks. The majority (70%) of the patients had primary refractory disease. The overall response rate was 75% with 34% complete remission and 40% partial remission, and a median PFS and OS of 5.6 months and 22.4 months, respectively. The predominant adverse effects were peripheral sensory neuropathy (43%), fatigue (40%), nausea (35%), neutropenia (19%), diarrhea (18%), and pyrexia (16%) [18].

Brentuximab was also found to enable some patients to proceed to allogeneic stem cell transplantation and did not adversely affect stem cell engraftment, graft versus host disease, or survival [19].

Brentuximab was also effective in patients who relapsed after allogeneic stem cell transplantation, demonstrating a 50% overall response rate and a median progression-free survival of 7.8 months [20].

### 7.3. Targeting CD20: Rituximab

CD20 antigen is highly expressed by the reactive B cells in the microenvironment of HL [21]. In a pilot study, 22 patients with relapsed classical HL were treated with six weekly doses of rituximab. Five patients (23%) achieved partial or complete remission and eight additional patients had stable disease (SD). Clinical remission was observed in patients regardless of CD20 expression by HRS cells and was limited in patients whose disease was confined to the lymph nodes [22]. In a phase II study, rituximab was used in combination with gemcitabine in 33 patients of RR-HL, out of which 55% already had a prior ASCT. Objective responses occurred in 16 patients (48%). The median duration of failure-free survival was 2.7 months. Grade 3 or 4 toxic effects included neutropenia and thrombocytopenia [23].

### 7.4. Targeting CD25: Daclizumab

CD25, an interleukin-2 receptor (IL-2R) alpha subunit, is expressed in adult T-cell leukaemia, cutaneous T-cell lymphoma, ALCL, and hairy cell leukaemia and on Reed-Sternberg and associated polyclonal T-cells in HL. In a recent phase II trial, daclizumab conjugated with the radionuclide yttrium-90 (90Y-daclizumab) was investigated in 30 relapsed/refractory HL patients. Twelve patients achieved CR, seven achieved PR, and five had SD. The main side effects were haematological, with prolonged thrombocytopenia, as well as three patients who developed a myelodysplastic syndrome following treatment [24].

### 7.5. Systemic Chemotherapy

Systemic chemotherapy is often used as a salvage option in patients with RR-HL failing ASCT. However the downside with these regimens includes modest response rates and shorter duration of response ranging from 6 to 8 months.

Various agents like vinblastine [25], vinorelbine [26], gemcitabine [27–29], and bendamustine have shown some antitumor activity either as a single agent or in combinations.

Gemcitabine in combination with vinorelbine has been tested in a limited number of patients with HL after ASCT, with an encouraging response rate (75%, 6 of 8 patients) [28]. Bartlett et al. reported the results of a combination of gemcitabine, vinorelbine, and pegylated liposomal doxorubicin (GVD) in patients with recurrent HL, both prior to and for relapse after ASCT [29]. The response rate among 39 patients receiving GVD for relapse after ASCT was 75% (17% CR), and median event-free survival was 8.5 months. However, the combination therapy in this setting was associated with significantly more myelosuppression than observed in patients who had not previously undergone ASCT.

Bendamustine is a mechlorethamine derivative with alkylating and antimetabolite properties. It showed marked antiproliferative and proapoptotic effects on HL cell lines [30]. Moskowitz et al. reported the activity of single-agent bendamustine in RR-HL that previously failed ASCT, allogeneic stem cell transplantation (Allo-SCT), or ineligible for transplant [31]. In this phase II study, bendamustine was administered at a dose of 120 mg/m^2^ for two consecutive days, every 28 days, for up to maximum of 6 cycles. Of evaluable 34 patients, there were 12 complete responses (CRs) (33%) and 7 partial responses (PRs) (19%) for an ORR of 56%. Further studies of both single-agent bendamustine and in combination with any other chemotherapy are warranted in RR-HL patients.

### 7.6. Novel Agents

#### 7.6.1. Bortezomib

NF-*κ*B is a transcription factor responsible for cell proliferation and antiapoptosis in HL [32]. Bortezomib (Velcade), a proteasome inhibitor, inhibits NF-*κ*B pathway. The cancer and leukemia group B (CALGB) conducted phase II clinical trials (CALGB 50206) evaluating bortezomib monotherapy in RR-HL. Disappointingly, no clinical activity was observed in treated patients [33]. In another study, bortezomib was used in combination with gemcitabine in 18 patients with relapsed HL. The overall response rate was 22% with more treatment related liver toxicity [34]. Overall, bortezomib did not show encouraging results in RR-HL.

#### 7.6.2. Histone Deacetylase Inhibitors

Histone deacetylases (HDACs) are crucial in cell proliferation, apoptosis, angiogenesis, and immune regulation. Their alteration has been found to be associated with various malignancies including HL.

Vorinostat, is a potent inhibitor of class I and II HDAC. In a phase II clinical trial evaluating the safety and efficacy of vorinostat in refractory/relapsed HL, a total of 25 patients were treated with vorinostat at 200 mg given orally twice per day for 14 days every 21-day cycle. The activity of vorinostat was modest and only one patient achieved a partial remission (PR) [35].

Panobinostat, is a potent pan-HDAC inhibitor and more potent than vorinostat in lymphoma preclinical models. A phase II study of panobinostat in RR-HL showed encouraging clinical activity with ORR 27%, 5 patients achieving CR, and 30 patients achieving PR with median PFS of 6.1 months [36]. Currently, the role of panobinostat in maintenance treatment of patients after high-dose chemotherapy is being evaluated in a phase 3 trial.

#### 7.6.3. Mammalian Target of Rapamycin Inhibitors

The phosphatidylinositol 3-kinase (PI3K) Akt/mTOR signalling pathway is one of the most aberrantly activated survival pathways in cancer, making it an important target for drug development [37]. Everolimus, an mTOR inhibitor, has demonstrated antiproliferative effect in several solid tumor and hematologic malignancies including HL. A phase II trial evaluated the clinical activity and toxicity of everolimus in patients with heavily pretreated RR-HL (median of 6 prior therapies and 84% had prior HDCT-ASCT). Of 19 patients, one patient achieved CR and 8 patients achieved PR resulting in ORR of 47%, although median time to response was only 7.2 months [38]. A phase I clinical trial combining the HDAC inhibitor panobinostat with the mTOR inhibitor everolimus is currently enrolling patients with non-Hodgkin's lymphoma (NHL) and HL.

#### 7.6.4. Lenalidomide

Lenalidomide, an immunomodulatory drug, has emerged as a promising therapeutic option in patients with RR-HL. In a phase II study, 38 patients were treated with lenalidomide at a dose of 25 mg/day on days 1–21 of a 28-day cycle out of which 87% patients had undergone prior stem cell transplantation. The responses were 1 complete remission (CR), 6 partial remissions (PRs), and 5 patients with stable disease (SD) for ≥6 months resulting in an objective overall response rate (ORR) of 19% and a cytostatic ORR of 33%. The treatment was well tolerated with the most common grade 3/4 AEs being neutropenia (47%), anemia (29%), and thrombocytopenia (18%) [39].

#### 7.6.5. Heat Shock Protein 90 Inhibitors

Heat shock proteins are cellular chaperone proteins required for essential housekeeping. Similarly HSP90 has been found to be overexpressed in primary and cultured HL cells [40]. Schoof et al. showed that inhibition of HSP90 by either geldanamycin derivative 17-AAG or RNA interference in HL cells led to decrease in cell proliferation and inhibition of STAT1, STAT3, STAT5, and STAT6 tyrosine phosphorylation possible secondary to reduced protein expression of Janus kinase (Jaks) [41]. HSP90 may be a promising target in patients with RR-HL.

#### 7.6.6. EBV Specific CTL Therapy

Nearly 30–40% of HL patients are known to be EBV positive, making the use of EBV-targeted therapy an attractive option. EBV specific cytotoxic T lymphocytes (CTL) can be generated* in vitro* and then given to the patients with the intent of targeting specific EBV-infected cancer cells. Lucas et al. demonstrated the clinical efficacy of allogeneic EBV-specific CTLs in EBV-positive RR-HL which had previously failed HDC-ASCT. Significant clinical activity was observed following allogenic CTLs infusion despite a lack to detect donor chimerism. In addition, in a limited number of patients, better clinical responses were observed when fludarabine was administered prior to CTLs infusion [42]. However, this therapy is still investigational.

#### 7.6.7. Farnesyltransferase Inhibitors

Farnesyltransferase is 1 of 3 prenyltransferases used by normal and malignant cells to catalyze covalent attachment of prenyl groups to *∼*300 polypeptides in the human proteome. Farnesyltransferase inhibitors (FTIs) diminish cell proliferation and induce apoptosis in a variety of preclinical models [43].

In a phase II study, tipifarnib was tried on 93 patients of relapsed non-Hodgkin's lymphoma and RR-HL (20% = 19/93). The overall response rate was 21% in RR-HL with 2 complete responses and 2 partial responses. The median time to progression and median overall survival was 3.6 months and 14.8 months, respectively, in all patients. Tipifarnib was well tolerated; the main adverse effects were mainly myelosuppression. More studies are required to test its efficacy in RR-HL either as a single agent or in combination with other drugs [44].

## 8. **Treatment Approach**

Although there are many treatment strategies, the standard approach is still lacking. Crump [9] devised a treatment approach for such patients which seems to be optimal. Patients having localized relapse should be considered for involved- or extended-field radiation, if not previously irradiated. Allogeneic stem cell transplant is the only strategy that allows achieving long-time survival. Reduced-intensity allogeneic transplantation should be considered in suitable patients (HLA-matched donor, young age, and good performance status) if relapse occurs >6 months after ASCT. For patients who are not candidates for allogeneic stem cell transplant, single-agent or combination chemotherapy with gemcitabine or vinorelbine may be tried with some beneficial effects. Patients with early relapse (<6 months after ASCT) and those refractory to chemotherapy may be considered for targeted therapies or participation in clinical trials. Selected patients who relapse >5 years from ASCT may be taken up for a second autologous transplant, but the long-term benefits are not known. The use of novel biological and targeted therapies is a promising approach but where to put them in treatment algorithm is still not clear. As the armamentarium for the treatment of patients with RR-HL continues to expand, we would be left with more complexities than comforts. For better understanding, further studies and randomized clinical trials are required.

## Figures and Tables

**Table 1:** Selected clinical studies on treatment options in patients with relapsed/refractory Hodgkin's lymphoma after autotransplant.

Author	Agent	Study design	Number of patients	Prior ASCT	Response	Median duration of response	Toxicity
Younes et al. [18]	Brentuximab vedotin	II	102	102 70% PRD	ORR 75% CR 34% PR 40%	PFS 5.6 months OS 22.4 months	PSN, fatigue, nausea, neutropenia, diarrhoea, and pyrexia

Younes et al. [22]	Rituximab	N/A	22	NR	ORR 22% CR 1, PR 4	8.7 months	NR

Oki et al. [23]	Rituximab + gemcitabine	II	33	55%	ORR 48% CR 5, PR 11	FFS 2.7 months	Gr 3-4 neutropenia and thrombocytopenia

O'Mahony et al. [24]	Daclizumab	II	30	NR	CR 12, PR 7, SD 5	N/A	Thrombocytopenia

Little et al. [25]	Vinblastine	N/A	17	17	ORR 59% CR 12% PR 47%	OS 38.8 months	Fever and neutropenia

Devizzi et al. [26]	Vinorelbine	N/A	24	NR	ORR 45%	N/A	N/A

Venkatesh et al. [27]	Gemcitabine	II	27	18	PR 22% SD 52%	6.4 months	Gr 3 thrombocytopenia, neutropenia, and anemia

Spencer et al. [28]	Vinorelbine + gemcitabine	II	8	8	ORR 75%	N/A	N/A

Bartlett et al. [29]	Gemcitabine + vinorelbine + pegylated liposomal doxorubicin	II	91	40	ORR 75% RR in prior ASCT Pts 70%	8.5 months in prior ASCT	Gr 3-4 thrombocytopenia neutropenia, and mucositis

de Filippi et al. [30]	Bendamustine	II	34	75%	ORR 56% CR 33% PR 19%	5 months	Gr 3-4 thrombocytopenia, anemia, and infections

Mendle et al. [34]	Bortezomib + gemcitabine	II	18	NR	ORR 22%	NR	Gr 3 elevation of transaminase enzyme

Kirschbaum et al. [35]	Vorinostat	II	25	NR	ORR 4%	PFS 4.8 months	Gr 4 anemia and lymphopenia Gr 3 thrombocytopenia

Younes et al. [36]	Panobinostat	II	129	NR	DCR 74% CR 5 PR 30	PFS 6.1 months	Gr 3-4 anemia, neutropenia, and thrombocytopenia

Johnston et al. [38]	Everolimus	II	19	84%	ORR 47% CR 1 PR 8	TTP 7.2 months	Gr >3 pulmonary toxicity

Fehniger et al. [39]	Lenalidomide	II	36	87%	ORR 19% CR 1 PR 6	NR	Gr 3-4 anemia and neutropenia thrombocytopenia

Georgakis et al. [40]	Tipifarnib	II	19	NR	ORR 21% CR 2 PR 2	TTP 3.6 months OR 14.8 months	Gr 3-4 anemia, neutropenia, thrombocytopenia, and fatigue

ASCT: autologous stem cell transplant; ORR: overall response rate; DCR: disease control rate; CR: complete response; PR: partial response; PFS: progression-free survival; FFS: failure-free survival; TTP: time to progression; Gr: grade; DOR: duration of response; NR: not reported.

## Abbreviations

HL: Hodgkin lymphoma
RR-HL: Relapsed/refractory Hodgkin lymphoma
ASCT: Autologous stem cell transplant
PET: Positron emission tomography
HDCT: High-dose chemotherapy
FFTF: Freedom from treatment failure
SRT: Salvage radiotherapy
OS: Overall survival
TRM: Treatment related mortality
CR: Complete remission
Allo-SCT: Allogeneic stem cell transplant
PFS: Progression-free survival
DFS: Disease-free survival
GvL: Graft versus lymphoma
NRM: Nonrelapse mortality
RIC: Reduced-intensity conditioning
MTD: Maximum tolerated dose
HRS: Hodgkin Reed Sternberg
SD: Stable disease
PR: Partial remission
ALCL: Anaplastic large cell lymphoma
ORR: Overall response rate
HATs: Histone acetyltransferases
HDACs: Histone deacetylase
NF-*κ*B: Nuclear factor kappa-light-chain-enhancer of activated B cells
CTLs: Cytotoxic T-lymphocytes
FTIs: Farnesyltransferase inhibitor.
